# Phytochemical Screening of *Rosmarinus officinalis* L. as a Potential Anticholinesterase and Antioxidant–Medicinal Plant for Cognitive Decline Disorders

**DOI:** 10.3390/plants11040514

**Published:** 2022-02-14

**Authors:** Majid Rasool Kamli, Abeer Abdullah M. Sharaf, Jamal S. M. Sabir, Irfan A. Rather

**Affiliations:** 1 Department of Biological Sciences, Faculty of Science, King Abdulaziz University, Jeddah 21589, Saudi Arabia; jsabir@kau.edu.sa; 2 Center of Excellence in Bionanoscience Research, King Abdulaziz University, Jeddah 21589, Saudi Arabia; 3 Jeddah Regional Lab, The Saudi Ministry of Health, Jeddah 21482, Saudi Arabia; absharaf@moh.gov.sa

**Keywords:** Alzheimer’s disease, *Rosmarinus officinalis*, rosemary, acetylcholinesterase, molecular docking, rosmarinic acid, carnosic acid

## Abstract

The inhibition of acetylcholinesterase (AChE) by cholinergic agents has been promoted as a potent strategy for treating and managing cognitive decline disorders. A wide range of natural products has long been used as potential sources or formulations of cholinergic inhibitors. Therefore, this study aimed to evaluate different *Rosmarinus officinalis* L. (*R. officinalis*) extracts for their AChE inhibitory activity using galanthamine as a standard AChE inhibitor. In this study, the ethyl-acetate extract (at a concentration of 250 µg/mL) exhibited the greatest inhibitory effect against AChE with significant inhibition of 75%, comparable to the inhibitor galanthamine with an inhibition of 88%. Kinetic analysis revealed that the extracts could induce a mixed type of inhibition, as observed in the case of galanthamine, with the highest increased *Km* and decreased *Vmax* values in the ethyl acetate extract. The antioxidant potential of the three extracts tested was found to be in the order of ethyl-acetate > ethanol > aqueous, with IC_50_ values of 272 µg/mL, 387 µg/mL, and 534 µg/mL, respectively. Ethyl-acetate was found to have the highest total phenolic content in all extracts. Further, in silico study showed structural binding characterization of rosmarinic acid and carnosic acid with human AChE enzyme. Rosmarinic acid showed strong binding and formed two hydrogen-bonding interactions with Ser-293 and Arg-296. In light of this, the ethyl-acetate extract of the plant may provide some novel potential pharmacological leads for treating and managing cognitive disorders such as Alzheimer’s.

## 1. Introduction

Alzheimer’s disease (AD) is a neurodegenerative disorder that causes atrophy and brain cells to die—described as a continuous decline in thinking, behavioral, and social activities [1]. AD primarily affects people older than 65 years of age. Globally, about 55 million people are estimated to have dementia in 2020. This number will almost double over the next 20 years, reaching 78 million in 2030 and 139 million in 2050. The majority of the increase will occur in developing countries. Sixty-one percent of people with dementia live in low- and middle-income countries, but by 2050, that number will increase to seventy-one percent. China, India, South Asia, and the western Pacific are experiencing the fastest growth of AD in the elderly population. The prevalence of AD is estimated to increase by many folds in the coming two decades, with 47 million people currently suffering from it worldwide [2]. AD affects about 4 million people in India and approximately 5.8 million people in the United States [3]. There are approximately 50 million people worldwide with dementia, between 60% and 70% of whom are estimated to have AD [3].

In the current state of research, there is no permanent cure for AD or method to alter the mental process [1]. In addition, as the disease progresses, the brain may suffer from severe complications such as dehydration, malnutrition, or infection that may cause death. To mitigate these symptoms, some medications may temporarily help patients to maintain independence for a limited time [1].

In addition to the protein tau, the accumulation of the protein amyloid plays a significant role in AD development. However, the AD diagnosis is made when a person’s cognitive functioning has declined sufficiently to meet dementia criteria [4]. However, AD is diagnosed when a person has sufficient cognitive decline to meet the criteria for dementia. One of the major reasons for the cognitive decline in AD is the degenerating cholinergic neurons in the basal forebrain and the related cholinergic neurotransmission loss in the cerebral cortex and other parts of the brain. Due to sufficient evidence for the deficit cholinergic neurotransmission in AD, the most promising approach promoted for AD treatment includes enhancing acetylcholine (ACh) levels in the brain [5]. Several strategies are used to improve cholinergic neurotransmission [6,7]. Among them, the most successful approach used so far is the cholinergic hypothesis [5]. Cholinergic receptors are stimulated for the enhancement of acetylcholine or to improve ACh’s ability to access the neuronal synaptic cleft by inhibition of acetylcholinesterase (AChE) induced ACh hydrolysis through the use of AChE inhibitors (AChEIs) [8,9]. The acetylcholine-hydrolyzing enzyme AChE is located in nerve tissues and is involved in the termination of nerve impulse transmission by catalyzing the hydrolysis of the neurotransmitter acetylcholine [10]. AChEIs have been shown to promote an increased concentration of ACh and enhance the duration of ACh action at the synapse [11]. Therefore, cholinesterase inhibitors are considered one of the effective medications for the symptomatic treatment of AD [12]. In this context, Tacrine, donepezil, and rivastigmine are a few AChE inhibitors approved by the U.S. Food and Drug Administration (FDA) [13].

As a result, therapeutic strategies for AD treatment were mainly focused on AChEIs, which led to various synthetic anti-AD drugs such as Galanthamine, Tacrine, Donepezil, etc. However, these drugs were found to have many restrictions due to their shorter half-lives and adverse side effects such as vomiting, nausea, anorexia, and fatigue, with some even exhibiting hepatotoxicity [14,15]. Therefore, scientists worldwide have been exploring alternative strategies to manage this age-related disease, including using herbal medicines as efficient anti-AD drugs or scaffold molecules. [16,17,18]. The probability of decreasing the AD-induced brain degeneration with natural treatments has made this popular and has drawn scientists’ awareness. More importantly, most synthetic anticholinesterase drugs have originated from plant-based molecules, including major bioactive substances such as indole, steroids, alkaloids, glycosides, coumarins, phenylpropanoids, and terpenoids [10].

*Rosmarinus officinalis* (family Lamiaceae), commonly known as rosemary, is one of the most popular perennial culinary herbs cultivated worldwide [19,20]. Fresh and dried rosemary leaves have been used in food preparation and herbal teas for their characteristic aroma. As a natural antioxidant, rosemary extracts are routinely used as a preservation agent in perishable foods [21,22]. As a natural antioxidant, the European Union has approved rosemary extract (E392) for food preservation. It is also used in traditional medicine in many countries, growing wild even outside its native Mediterranean. Aside from its antibacterial, anticancer, anti-diabetic, anti-inflammatory, antinociceptive, antioxidant, and antithrombotic properties, rosemary has been shown to effectively treat cognitive deficiency, reduce thirst, and improve hepatic function [23,24,25,26,27]. *R. officinalis* oils serve as natural components in perfumes, foods, and pharmaceuticals. Furthermore, at the folklore level, rosemary has been actively used for memory enhancement as well as reducing age-related memory loss in humans [28,29,30]. However, scientific data regarding its ability to inhibit anticholinesterase activity are entirely lacking. Therefore, considering the alarming increase in AD, this perennial culinary herb was evaluated for anticholinesterase activity.

## 2. Results

### 2.1. Functional Group Determination and Total Phenolic Content of the R. officinalis Extracts

**Figure 1:**
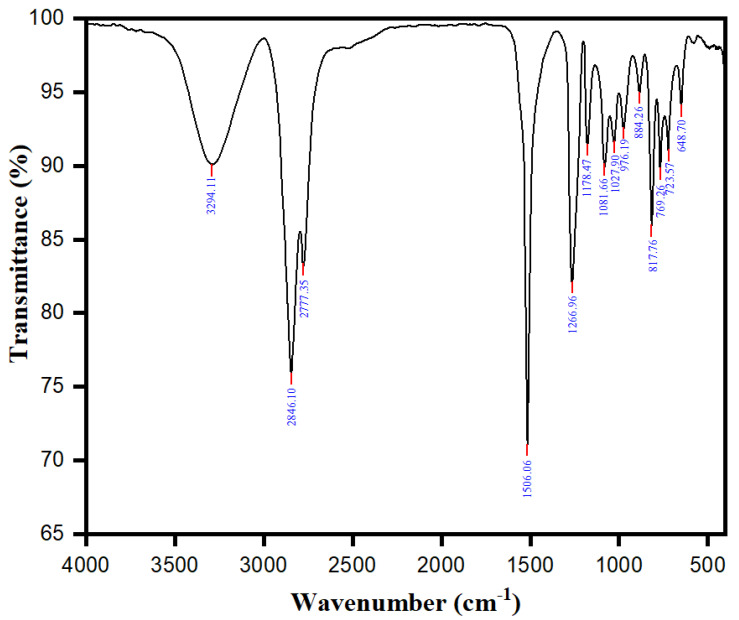
FTIR spectra of *R. officinalis* extract.

**Figure 2:**
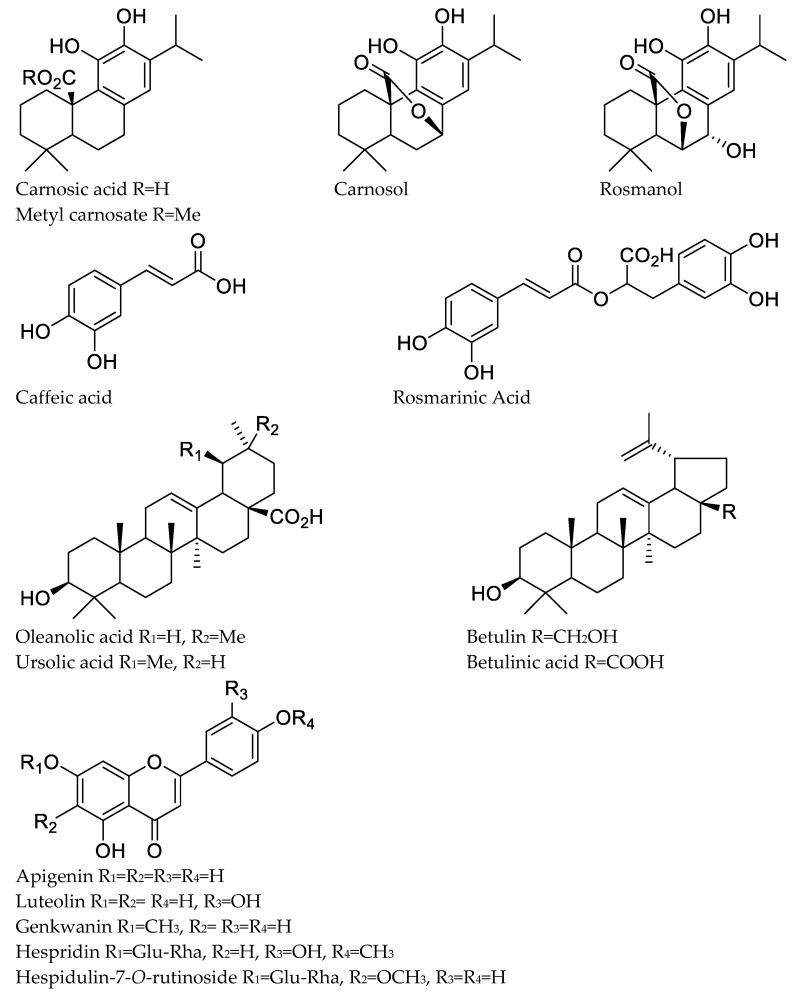
Major chemical constituents present in the *R. officinalis* extract.

**Figure 3:**
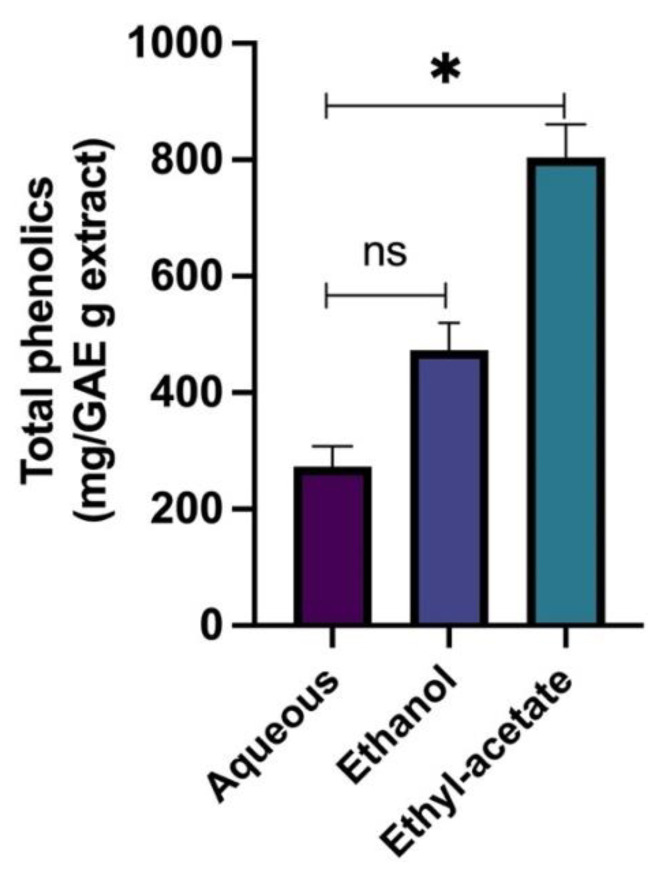
Total phenolic content of different extracts of *R. officinalis.* Data represent the mean of the results obtained from three independent measurements with their standard deviations as error bars. Data were analyzed using *t*-test and one-way ANOVA. * Indicates significance at *p* < 0.05 when compared to the aqueous (*p* value = 0.0149), ns indicates not significant (*p* value = 0.0606).

One of the most commonly used techniques for distinguishing functional groups is Fourier Transform Infrared (FTIR) spectroscopy. The FTIR spectra (in the range of 400–4000 cm^−1^) and the characteristic bands observed in the *Rosmarinus officinalis* leaf extract are shown in Figure 1 and supplementary Table S1, respectively. The main bands found for the total *R. officinalis* extracts are assigned to the presence of hydroxyl group (O-H), carboxylic group (COOH), carbonyl group (C=O), (C-H), and (C=C), whose corresponding peak values are presented in supplementary Table S1. All these characteristics correspond to various flavonoids and phenolic compounds present in the *R. officinalis* extracts (Figure 2). The principal bioactive constituents of rosemary leaves are rosmarinic acid and carnosic acid, which have antioxidant, anti-inflammatory, and anti-carcinogenic properties. The other major chemical constituents in the *R. officinalis* extract included flavonoids, terpenoids, and common organic acids (Figure 2). The total phenolic content of various extracts of *R. officinalis* was estimated by the Folin–Ciocalteu method and was represented as gallic acid equivalents (GAE/g extract). Among them, a significant amount of phenolic content of 804 GAE/g extracts was found in ethyl-acetate extract (EtOAc; *p* = 0.0149) followed by ethanolic (473 GAE/g) and aqueous (273 GAE/g), as shown in Figure 3. Data are the mean of the results obtained from three separate measurements with their standard deviations as error bars.

### 2.2. Free Radical Scavenging Potential of the Rosemarinus Officinalis Extracts

**Figure 4:**
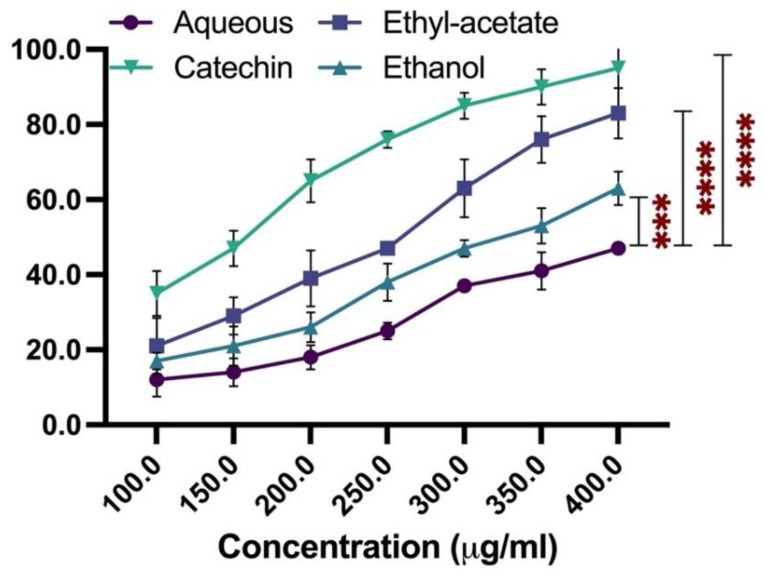
DPPH radical scavenging activity of the extracts of *R. officinalis*. Catechin was used as a positive control. The presented data represent the mean of three independent measurements with an average 5–7% error. Data were analyzed using one-way ANOVA *** and **** indicate significance at *p* < 0.05 when compared to aqueous with *p* values 0.006 and 0.001, respectively.

The extracts’ antioxidant activity was determined using DPPH assay in which various concentrations of the *R. officinalis* extract were added to DPPH. Further, the amount of left out DPPH was estimated at 30 min by measuring absorbance at 520 nm. Next, DPPH scavenging activity was calculated for each plant extract concentration using the absorbance data accumulated and % inhibition of DPPH scavenging. Following this, IC_50_ values were determined for DPPH free radical scavenging as the plant extract concentration with an ability to bring 50% of the original activity. It was found that the (EtOAc) exhibited the most significant antioxidant activity, with an IC_50_ value of 272 µg/mL (*p* = 0.001), followed by ethanolic (*p* = 0.006) and aqueous extracts (*p* =0.006) with IC_50_ values of 387 and 534 µg/mL, respectively. For the standard inhibitor catechin, the IC_50_ value was found to be 173 µg/mL (Figure 4).

As expected, the observed antioxidant activity of the extracts exhibited a strong correlation with their total phenolic content, as EtOAc, with the highest TPC, showed the most significant antioxidant activity. The cytotoxic effect of ethyl-acetate extract on MCF-7 cells was evaluated using MTT assay and is shown in supplementary Figure S1.

### 2.3. Inhibition of Acetylcholinesterase by the Extracts

**Figure 5:**
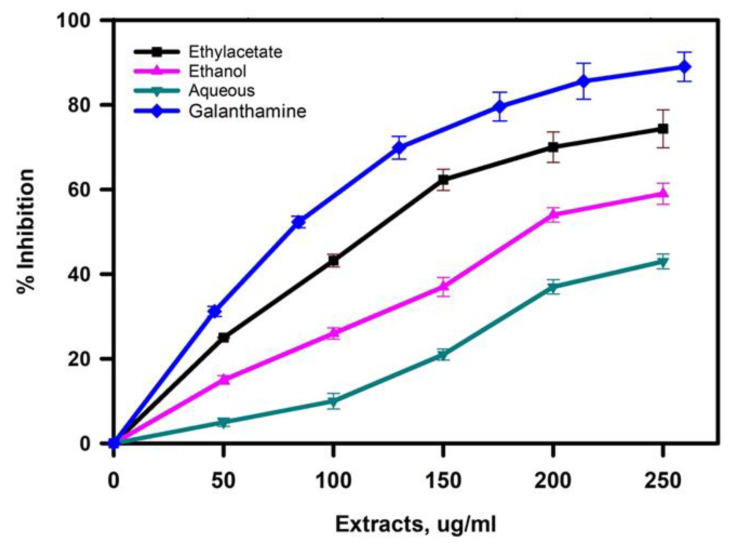
Acetylcholinesterase inhibition potential of extracts of *R. officinalis*. Results presented are the mean of three independent measurements.

Extracts of *R. officinalis* were evaluated for AChE inhibitory by using modified Ellman’s method. Figure 5 and Table 1 represent the percentage inhibition of AChE activity by different concentrations of the *R. officinalis* extracts and each extract’s IC_50_ values. For the positive control, galanthamine, a standard AChE inhibitor, was used and exhibited an IC_50_ value of 4.73 ± 0.13 µg/mL. IC_50_ values were lowest for ethyl-acetate extract followed by ethanolic and aqueous extract with respective values of 101.23, 202.34, and 247 µg/mL. Interestingly, the antioxidant activity of the extracts with their phenolic content was also observed in the same manner. Further, the free radical scavenging ability, i.e., ethyl-acetate’s antioxidant activity, was highest, followed by ethanolic and aqueous extracts, as shown in Figure 3.

### 2.4. Kinetics of AChE Inhibition Observed by the R. officinalis Extracts

**Figure 6:**
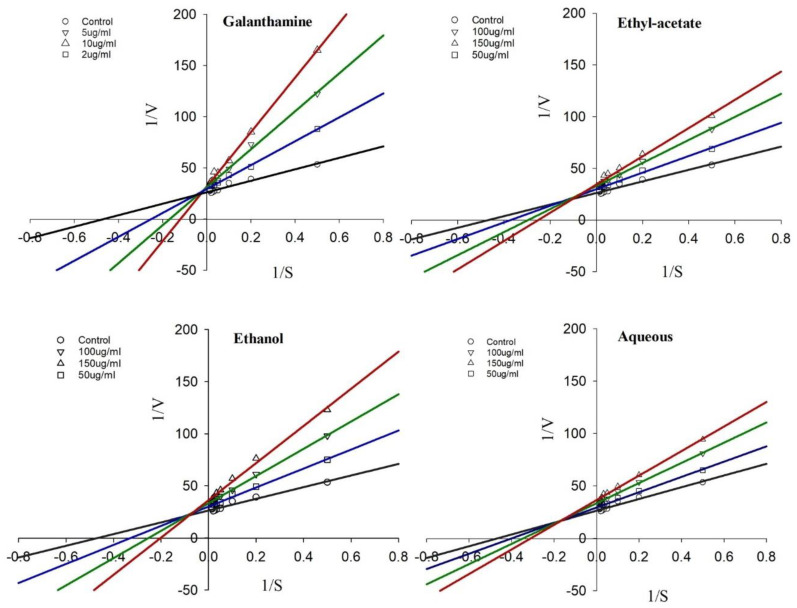
Kinetics of acetylcholinesterase inhibition by *R. officinalis*. Lineweaver–Burk plot of inhibition of acetylcholinesterase by different extracts of *R. officinalis.* Representative data are the average of 3 independent measurements.

The lack of data on AChE inhibition kinetics by medicinal plant extracts has been observed in traditional therapeutic systems. However, very few studies carried out in this direction have shown that medicinal plant extracts may show enzyme inhibition kinetics close to reported synthetic AChEIs. In this context, AChE enzyme inhibition kinetics of *R. officinalis* extracts, reported for the first time in this study, were determined from Lineweaver–Burk plots (Figure 6). Kinetic parameters Km and Vmax from the inhibition kinetic curves were determined from the plotted graph’s trend line equations. The kinetic parameter values obtained at each concentration of the extracts have been summarized in Table 2. The extracts exhibited a mixed type of inhibition, as indicated by the intersection of trend lines with each other on the left side above the X-axis of the kinetic plot (Figure 6).

### 2.5. Molecular Docking of Rosmarinic Acid and Carnosic Acid with Human AChE 

**Figure 7:**
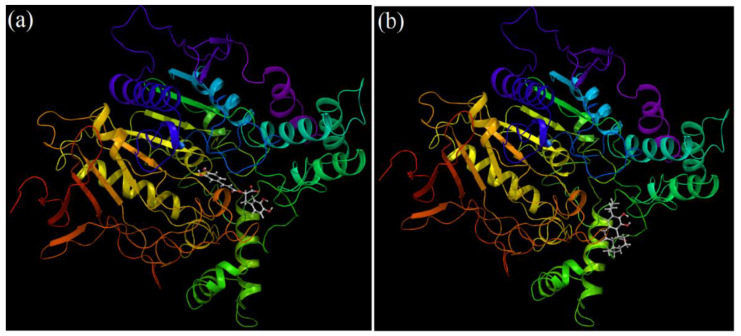
Docking complex of cholinesterase with (**a**) rosmarinic acid and (**b**) carnosic acid.

**Figure 8:**
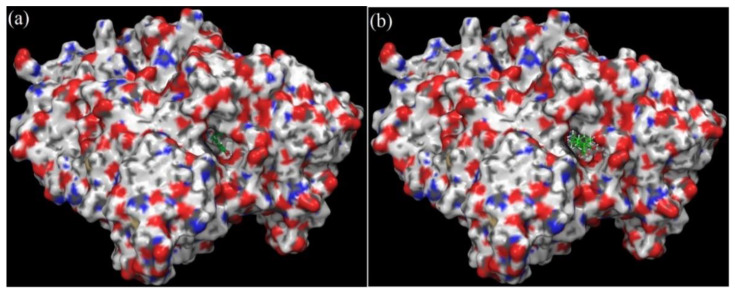
Surface structure of cholinesterase with (**a**) rosmarinic acid and (**b**) carnosic acid.

**Figure 9:**
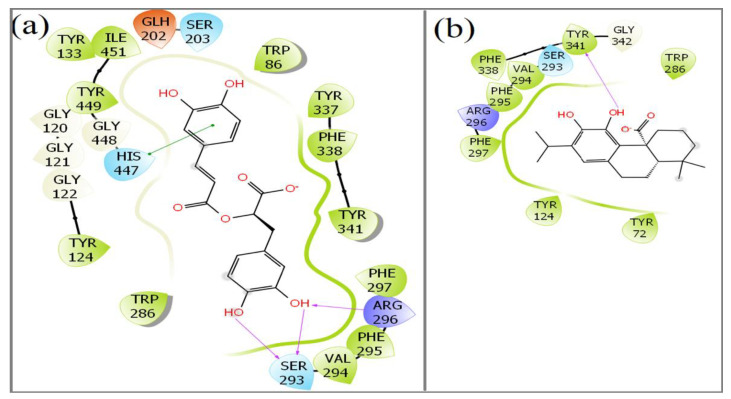
Interaction of amino acid residues of acetylcholinesterase with (**a**) rosmarinic acid and (**b**) carnosic acid.

Both the compounds docked stably into the ligand pocket of the human AChE enzyme (Figure 7). The surface representation of docking complexes of both the ligands with AChE is shown in Figure 8. The observed docking scores for rosmarinic acid and carnosic acid are −8.25 and −5.10, respectively. Rosmarinic acid formed two hydrogen-bonding interactions with Ser-293. Moreover, additional hydrogen bond interaction was observed with Arg-296. Furthermore, one π–π interaction was also displayed by His-477 with rosmarinic acid (Figure 9a). On the contrary, carnosic acid showed only one hydrogen interaction with Try-341 (Figure 9b). *Rosmarinus officinalis* ethyl-acetate leaf extract contains different significant phenolic compounds, including rosmarinic acid and carnosic acid, which were also confirmed by high performance liquid chromatography (HPLC) analysis as shown in Figure S2. The other major components identified were carnosol, methyl carnosate, rosmanol, genkwanin, camphene, borneol acetate, tricyclene, linalool, alpha-terpineol, and bornyl acetate.

## 3. Discussion

Sufficient shreds of evidence for deficit cholinergic neurotransmission in AD have promoted therapies designed to reverse this cholinergic deficit, mainly based on employing the AChEIs to inhibit AChE. AChEIs are considered one of the most effective medications used for the symptomatic treatment of AD by increasing cholinergic neurotransmission with moderate and short-lived but promising therapeutic effects [31,32]. These have been found to increase the concentration of Ach and prolong the duration of Ach action at the synapse. In addition, these AChE inhibitors exhibit antioxidant activity and modulate amyloid-β plaque formation. The FDA has approved a few AChE inhibitors, such as Tacrine, donepezil, and rivastigmine [33]. However, most of these inhibitors are associated with acute toxicity and low availability. Due to these reasons, there is still a need to explore and identify new AChEIs with reduced toxicity and high availability with enhanced penetrance to the nervous systems. In this context, natural sources, mainly plants, have been investigated to identify new AChEIs, and many plant-derived compounds have been identified as AChEIs with potential for AD treatment [24,34]. Traditional medicinal systems for the treatment of nervous and aging disorders have documented many useful medicinal plants. Further, organic solvent extracts of *R. officinalis* were prepared and assessed for their antioxidant and anticholinesterase activities.

Previous studies reported that antioxidant activity of different plants is mainly due to the presence of the phenolic compounds [35,36]. Since oxidative stress plays an essential role in AD, these phenolic compounds have been promoted as effective therapeutic agents for this disease. Therefore, our extracts showed higher phenolic content, as expected. The free radical scavenging potential of the *R. officinalis* extracts results infers that the potent antioxidant potential of the (EtOAc) of *R. officinalis* may be attributed to the plants’ phenolic compounds, including flavonoids, aromatic compounds, and tannins. Literature studies have shown that polyphenols can inhibit AChE, and most of the AChEIs have been derived from phenolic compounds [37]. However, other non-alkaloid compounds such as terpenoids, sterols, flavonoids, etc. cannot be ruled out, as several non-alkaloid AChEIs have also been reported [38,39]. As reported by other studies, the present results also imply that the *R. officinalis* extracts’ antioxidant activity could be due to the synergistic effects of different phenolic compounds depending on the concentration and structural chemistry of the phenolic compounds.

Acetylcholinesterase (AChE) and antioxidant activity studies of *R. officinalis* strongly indicate that the extract with the most increased antioxidant activity exhibited the highest AChE inhibitory activity. The analysis of results suggests that, probably, higher phenolic content of ethyl-acetate extract might be responsible for its most elevated AChE and antioxidant activity. Further, the kinetics of AChE inhibition in *R. officinalis* extract showed a mixed type of inhibition. As reported by other studies, the mixed type of inhibition by the extracts may be due to the binding of active molecules present in the extracts to the free enzyme of the enzyme-substrate complex at a site different from that of the substrate-binding site. Probably, this allosteric site has been observed to the peripheral anionic site (PAS). PAS has been reported at the gorge of the active site channel of AChE and has been shown to sequester acetylcholine, the natural substrate of AChE, during cholinergic transmission. Results obtained in our studies conform to other AChE inhibition studies wherein binding of inhibitor ligands has been shown to occur at PAS, which leads to some conformational change in the enzyme at its active site. Some studies have shown that the PAS blockage due to steric hindrances by the binding ligands has also been observed as a major factor responsible for AChE inhibition by the inhibitors. Altogether, as per the results obtained in inhibition kinetics of AChE, it can be expected that the inhibition of AChE by *R. officinalis* extracts might probably be due to their ability to bind PAS or the steric hindrance phenomenon, as observed in other earlier studies. Either of these two reasons cannot be excluded and may likely involve both mutually. In addition, the concentration-dependent inhibition kinetics of AChE in the presence of *R. officinalis* extracts is consistent with those observed in pure compound inhibitors [40]. Our results for galanthamine as a mixed inhibitor are uncommon, as it has been normally shown to be a competitive inhibitor. However, some other studies have shown it to be a mixed-type inhibitor [41]. The differences in experimental methodology may be responsible for this unusual behavior of galanthamine. AChE inhibition kinetic results indicated a putative mechanism by which the plant extracts can likely be used as an innovative therapeutic for cognitive decline disorders such as Alzheimer’s. The phytotherapeutic approach’s significant benefits are the vast scope of medicinal properties that each extract exhibits, whereas the inhibitor compounds are usually intended to act on a single target.

The phytochemical profile of *R. officinalis* extract reported many polyphenolic and flavonoid compounds. Among the polyphenolic compounds, rosmarinic acid and carnosic acid are essential constituents of *R. officinalis* [42]. Polyphenol compounds from many plant extracts are well known for anticholinesterase activity [43]. Carnosic acid and its major oxidized derivative, carnosol, protect lipids from oxidation in vitro, as determined by high-performance liquid chromatography–ultraviolet and luminescence imaging. Both compounds protected linolenic acid and monogalactosyldiacylglycerol from the effects of singlet oxygen and hydroxyl radicals [44]. Thus, phenolic diterpenes carnosol is one of the significant compounds found in *R. officinalis* extract and is well-known for its antioxidant properties [45]. Additionally, the *R. officinalis* extract contains some highly oxidized diterpenes such as rosmanol, isorosmanol, and dimethyl isorosmanol, formed from carnosic acid via enzymatic dehydrogenation and the action of activated oxygen [46]. Rosmarinic acid and carnosic acid were selected for the molecular docking analysis as these compounds are reported for their anticholinesterase activity and anti-neuropathic effects [47,48,49]. Our in silico structural binding studies suggest that both compounds have the potential to inhibit AChE activity with calculated dock scores along with AChE amino acid residue interactions, indicating that the rosmarinic acid seems to have higher inhibiting potential than carnosic acid.

## 4. Materials and Methods

### 4.1. Chemicals

Acetylcholinesterase, DPPH (2,2-diphenyl-1-picrylhydrazyl), ascorbic acid, and BHT (Butylated hydroxytoluene) were obtained from Sigma Aldrich, St. Louis, MO, USA. Chemicals used, such as sodium carbonate, hydrochloric acid, etc., were of analytical grade, and organic solvents such as ethanol and ethyl-acetate used for extraction were purchased from Merck Life Sciences, Bengaluru, India.

### 4.2. Collection of Plant Material and Preparation of Extract

*R. officinalis* plant was collected from Botanical Garden, Srinagar, Jammu and Kashmir, India and was authenticated at the Department of Botany, University of Kashmir. After drying the collected plant material in the shade at a temperature of 30 ± 2 °C, it was crushed in a blender, and the powdered material was filtered by using a sieve of about 0.3 mm aperture size. The powdered material was extracted with the organic solvents ethyl-acetate, ethanol, and water for 48 h by using a Soxhlet extractor. The extracted material was concentrated and was stored at −80 °C for future use.

### 4.3. Estimation of Total Phenolic Content of the Prepared Rosmarinus Officinalis L. Extracts

For this, the Folin–Ciocalteu method was used, in which an extract sample volume of 1 mL was mixed with 2 mL of Folin–Ciocalteu, and both were kept together at 30 °C for 15–20 min in the dark. Following this, sodium carbonate 5 mL was mixed to the extract solution and incubated for 2 h. Finally, the absorbance was measured with a spectrophotometer at a wavelength of 745 nm. For estimation of the content, a standard calibration curve was prepared by using the standard gallic acid in the concentration range of 0.02–0.15 mg/mL, and measurements were recorded as mg gallic acid equivalent (GAE) per gram of the extracted sample.

### 4.4. Measurement of DPPH Radical Scavenging Activity

DPPH scavenging activity was measured by the modified method of Braca et al. [50]. Different concentrations (100–400) of the respective plant extract sample were mixed with DPPH and were vortexed. After proper mixing, the solutions were kept in the dark for 30 min at room temperature, and following this, the absorbance of the solution was measured at 520 nm. BHT was used as a standard (1.5 mg/mL). Percentage inhibition of the antioxidant activity was calculated by using Equation (1), and the values obtained were used for calculation of IC50 value, i.e., required concentration of extract sample for scavenging of 50% DPPH free radicals.

% inhibition = [(Ac−Ae)/Ac] × 100(1)

where Ac and Ae are absorbances of the solutions without extract sample and in the presence of catechins or the plant extracts, respectively.

### 4.5. Cell Culture and Cell-Viability Assay

Breast cancer cells (MCF-7) were cultured in Dulbecco’s Modified Eagle’s Medium (DMEM) supplemented with 10% fetal bovine serum (FBS) (HyClone Laboratories, Logan, UT, USA) and 1% penicillin: streptomycin (Invitrogen, Waltham, MA, USA) by incubation at 37 °C in a humidified atmosphere of 5% CO_2_ in 100 mm plates. The cells were seeded in 96-well plates for the cell viability assay at a density of 5 × 10^5^ cells per well. After the cells had adhered to the plate, the growth medium was replaced with fresh media, and the cells were cultured for 24 h with different concentrations of ethyl-acetate extract ranging from 0 to 250 ug/mL. For the control, PBS was used. Further, the cells were washed with PBS and treated with 50 μL of 5 mg/mL 3-(4,5-dimethylthiazol-2-yl)-2,5-diphenyl-2H-tetrazolium bromide (MTT) solution and incubated for 3 h at 37 °C to form the formazan crystals. After incubation, the media were removed from the cells. The cells were washed twice with PBS MTT formazan crystals, dissolved in 200 uL of DMSO, and mixed for 10 min to dissolve them completely. After that, absorbance was measured at 570 nm. The average number of live cells was calculated, and the experiments were performed in triplicate. The percentage of viable cells was estimated using the following formula.

(2) $$\mathrm{Cell}\;\mathrm{viability}\;\left(\%\right)=\frac{\left(\mathrm{optical}\;\mathrm{density}\;\mathrm{of}\;\mathrm{the}\;\mathrm{sample}-\mathrm{optical}\;\mathrm{density}\;\mathrm{of}\;\mathrm{control}\right)}{\mathrm{optical}\;\mathrm{density}\;\mathrm{of}\;\mathrm{control}\;}\times 100$$

### 4.6. Determination of AChE Enzyme Activity

The AChE enzyme activity was initiated via the modified method of Ellman [51]. In this procedure, 125 μL of AChE enzyme was added to a reaction mixture of 3 mL consisting of 50 mM Tris-HCl buffer, pH 7.3, with or without plant extract sample. After thorough mixing, the mixtures were incubated with each other for 15 min at room temperature. For initiating the reaction, 100 μL of 1 mM DTNB and 250 μL of 3.5 mM acetyl-thiocholine iodide were mixed. The reaction mixture was kept for 20 min and, following this, absorbance measurements were carried out at 412 nm in a UV/Vis Spectrophotometer. For control experiments, the enzyme was added after the addition of DTNB in the mixture. For positive control, galanthamine was used. All experiments were carried out in triplicate along with their respective controls. Percentage inhibition of the AChE activity was calculated using Equation (1) and the values obtained for calculating IC_50_ value.

### 4.7. Estimation of Inhibition Kinetic Parameters

To check the kind of inhibition exhibited by the plant extracts, the enzyme activity in the presence of extracted samples was measured in different concentrations of the substrate acetyl-thiocholine iodide (1–100 μM). The reaction mixtures were prepared by adding 125 μL of AChE enzyme combined with 0.05 M Tris-HCl (pH 7.3) and plant extract sample (in the concentration range of 50–150 µg/mL). For initiating the reaction, different concentrations of the substrate acetyl-thiocholine iodide with a concentration range of 1 to 100 μM were added, and the reaction was followed for 10 min. The kinetic curve obtained was evaluated for the determination of initial velocity as the slope of the curve. Lineweaver–Burk plots were made from the inhibition assays, and these plots’ Km and Vmax values were determined for each extract concentration [52].

### 4.8. Induced Fit Docking

Molecular docking of rosmarinic acid and carnosic acid with human AChE was performed. The docking simulation studies of these compounds were performed using Schrodinger 2017-4 suite. The Protein Data Bank (PDB) (http://www.rcsb.org/, access on 20 July 2020) was searched, and the three-dimensional structure of human AChE (PDB code: 6o52), having a resolution of 3.20 Å, was downloaded. The retrieved system was subjected to optimization and energy minimization using Schrodinger’s protein preparation wizard workflow. Further, the missing loops and side chains were also built. Similarly, ligand molecules were also prepared before performing the docking experiment. The two abovementioned ligands’ structures were drawn and converted to a three-dimensional structure using Maestro 11.4 (Maestro, version 11.4, Schrodinger, LLC, New York, NY, USA, 2017). LigPrep, version 3.1 (Schrodinger, LLC, New York, NY, USA, 2017) was applied for ligand preparation. The above-prepared protein and ligand molecules were subjected to Glide S.P. docking. The methodology is reported in detail [53,54].

### 4.9. Statistical Analysis

The data are presented as the mean and standard error of (S.E.M.) using GraphPad Prism 9.0 (GraphPad, San Diego, CA, USA). The one-way ANOVA technique was used to test whether the mean of the control group and the mean of all samples with one variable were significantly different. Multiple groups with two independent variables were analyzed using two-way ANOVA. An unpaired *t*-test was used to compare the means of only two groups. A *p*-value less than 0.05 was regarded as statistically significant.

## 5. Conclusions

In conclusion, the anticholinesterase activity of all the extracts, particularly ethyl-acetate, of *R. officinalis* validate their use in the traditional medicinal system for cognitive disorders such as Alzheimer’s. In addition, the potential anti-Alzheimer activity of the EtOAc of the plant highly warrants its future investigation to identify the active constituent molecules and assess their activity and safety under in vivo models. Further, *R. officinalis* extract contains many important chemical molecules such as rosemarnic acid and carnosic acid. In silico studies showed rosmarinic acid could interact with AChE amino acid residue and thus possess anticholinesterase activity. Therefore, the results of this study reinforce the potential therapeutic benefits of *Rosemarinus officinalis*. Further research and clinical trials are necessary to validate these findings and thereby uncover more evidence of its pharmacological effects.

## Figures and Tables

**Table 1:** IC_50_ values of DPPH free radical scavenging activity and acetylcholinesterase inhibition by *R. officinalis*.

Extract	DPPH Activity (µg/mL)	*p* Value ****	Anti-Cholinesterase Activity (µg/mL)	*p* Value ****
Catechin	173 ± 8	0.0001		0.0001
Galanthamine		0.0001	4.73 ± 0.13	0.0001
Ethyl-acetate	272 ± 7	0.0001	101.2 ± 5.5	0.0001
Ethanol	387 ± 11	0.0001	202.3 ± 8.7	0.0001
Aqueous	584 ± 8		247 ± 12	

**** indicate significant when *p* value <0.05.

**Table 2:** Kinetic parameters of acetylcholinesterase inhibition by extracts of *R. officinalis*.

Inhibitor	Concentration (µM; µg/mL)	*K*_m_ (µM)	*V*_max_
Galanthamine	0	2.14 ± 0.13	0.38 ± 0.09
	2	5.47 ± 0.17	0.29 ± 0.06
	5	9.76 ± 0.25	0.21 ± 0.07
	10	17.38 ± 0.33	0.15 ± 0.05
Ethyl-acetate	50	4.21 ± 0.28	0.32 ± 0.08
	100	7.19 ± 0.41	0.28 ± 0.10
	150	8.28 ± 0.63	0.23 ± 0.08
Ethanol	50	3.05 ± 0.21	0.35 ± 0.05
	100	4.65 ± 0.12	0.30 ± 0.06
	150	6.24 ± 0.53	0.28 ± 0.08
Aqueous	50	2.50 ± 0.09	0.34 ± 0.09
	100	3.67 ± 0.11	0.31 ± 0.04
	150	4.22 ± 0.29	0.27 ± 0.07

## Data Availability

Data is contained within the article and supplementary material.

## Supplementary Materials

The following supporting information can be downloaded at: https://www.mdpi.com/article/10.3390/plants11040514/s1, Supplementary Figure S1: Cytotoxicity of *R. officinalis* ethylacetate in MCF-7 cells; Supplementary Figure S2: HPLC high performance liquid chromatography of R. officinalis leaf ethyl acetate showing the peaks of the major components; Supplementary Table S1: FTIR spectra of R. officinalis L extract showing characteristic peaks and corresponding functional groups.
