# Supplementary material for: Phytochemical Screening of Rosmarinus officinalis L. as a Potential Anticholinesterase and Antioxidant–Medicinal Plant for Cognitive Decline Disorders
Source: Plants (Basel). 2022 Feb 14;11(4):514. doi: 10.3390/plants11040514 (PMC8877369; doi:10.3390/plants11040514)
Supplement: Supplementary file 1 [file plants-11-00514-s001.zip › plants-1525365-supplementary.pdf]

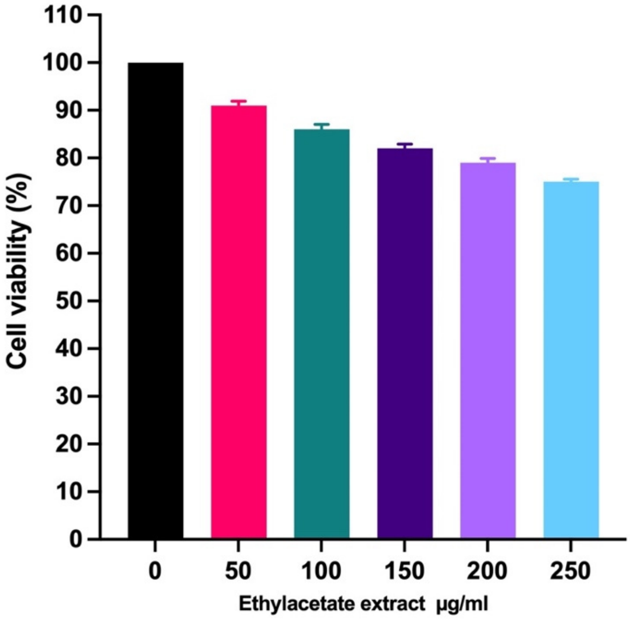

Supplementary Figure S1: Cytotoxicity of *R. officinalis* ethylacetate in MCF-7 cells.

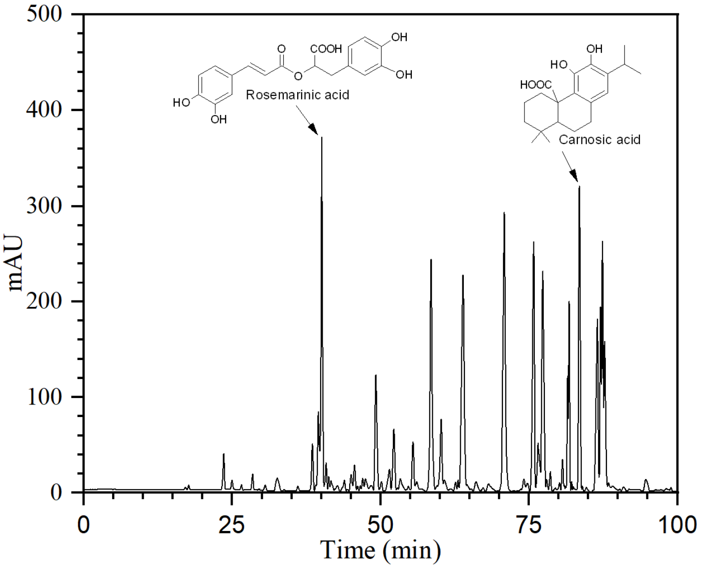

Supplementary Figure S2: HPLC high performance liquid chromatography of *R. officinalis* leaf ethyl acetate showing the peaks of the major components.

In brief, for HPLC the solvents used were of HPLC grade purchased from Merck Germany. High performance liquid chromatography (HPLC) analysis of *R. officinalis* L. ethyl acetate extract was performed for the quantification of main phenolic compounds, namely rosmarinic acid and carnosic acid by using Jasco 880-PU HPLC pump to deliver the mobile phase [S1]. A volume of 20μl was injected at the flowrate of 1mL/min to a Spherisorb ODS2 HPLC column (4.6mm x 250mm) and the chromatograms were acquired at 280 nm by absorption to detect the compounds.

S1. Moreno, S.; Scheyer, T.; Romano, C.S.; Vojnov, A. Antimicrobial and antioxidant activities of Argentinean Rosmarinus officinalis L. extracts. Free Radic Res. 40:223–231. 2006.

Supplementary Table S1. FTIR spectra of *R. officinalis* L extract showing characteristic peaks and corresponding functional groups.

No.	Peak (Wavenumber cm^{-1})	Intensity	Bond	Functional group assignment	Group frequency
1	3294.11	90.04	OH	OH stretching bands of alcohols (strong, broad)	3550-3200
2	2846.10	75.91	C-H	C-H stretching vibrations specific to CH_3 and CH_2 (alkane) (Medium)	3000-2840
3	2777.35	83.44	C-H	C-H stretching of aldehyde (medium)	2830-2695
4	1506.06	71.13	N-O	N-O stretching of nitro compound (Strong)	1550-1500
5	1266.96	82.12	C-O	C-O stretching of alkyl aryl ether (Strong)	1275-1200
6	1178.47	91.58	C-O	C-O stretching of ester, tertiary alcohol (strong)	1210-1124
7	1081.66	90.21	C-O	C-O stretching of primary alcohols (strong)	1085-1050
8	1027.90	91.70	C-O	C-O stretching of vinyl ether (Strong)	1075-1020
9	976.19	92.55	C=C	C=C bending of alkene (strong)	980-960
10	884.24	95.05	C-H	C-H bending of 1,2,4-trisubstituted or 1,3-disubstituted (strong)	900-700
11	817.76	86.05	C-H	C-H bending of 1,4-disubstituted or 1,2,3,4-tetrasubstituted (Strong)	900-700
12	769.26	89.92	C-H	C-H bending of monosubstituted (strong)	900-700
13	723.57	91.06	C-H	C-H bending of 1,2,3-trisubstituted (strong)	900-700
14	648.70	94.41	C-H	C-H out-of-plane bending vibrations from isoprenoids	700-600
